# 2-Chloro-*N*-(3-methyl­benzo­yl)benzene­sulfonamide

**DOI:** 10.1107/S1600536810016235

**Published:** 2010-05-08

**Authors:** P. A. Suchetan, B. Thimme Gowda, Sabine Foro, Hartmut Fuess

**Affiliations:** a Department of Chemistry, Mangalore University, Mangalagangotri 574 199, Mangalore, India; b Institute of Materials Science, Darmstadt University of Technology, Petersenstrasse 23, D-64287 Darmstadt, Germany

## Abstract

In the title compound, C_14_H_12_ClNO_3_S, the N—H bond is in an anti­periplanar conformation to the C=O bond. The dihedral angle between the two aromatic rings is 74.7 (1)°. The crystal structure features inversion-related dimers linked by N—H⋯O hydrogen bonds.

## Related literature

For background to our study of the effect of ring and side-chain substitutions on the crystal structures of *N*-aryl sulfonamides and for related structures, see: Gowda *et al.* (2009 ▶, 2010 ▶); Suchetan *et al.* (2010 ▶).
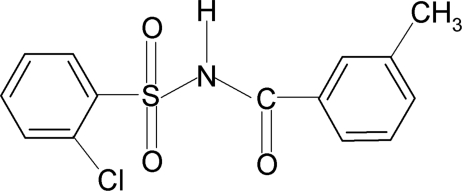


## Experimental

### 

#### Crystal data

C_14_H_12_ClNO_3_S
*M* *_r_* = 309.76
Orthorhombic, 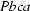

*a* = 12.4487 (8) Å
*b* = 13.4619 (8) Å
*c* = 17.455 (1) Å
*V* = 2925.2 (3) Å^3^
*Z* = 8
Mo *K*α radiation
μ = 0.41 mm^−1^
*T* = 299 K
0.30 × 0.30 × 0.20 mm

#### Data collection

Oxford Diffraction Xcalibur diffractometer with a Sapphire CCD detector
Absorption correction: multi-scan (*CrysAlis RED*; Oxford Diffraction, 2009 ▶) *T*_min_ = 0.887, *T*_max_ = 0.923
7216 measured reflections
2955 independent reflections
2422 reflections with *I* > 2σ(*I*)
*R*_int_ = 0.014

#### Refinement

*R*[*F* ^2^ > 2σ(*F* ^2^)] = 0.035
*wR*(*F* ^2^) = 0.095
*S* = 1.04
2955 reflections
186 parameters
1 restraint
H atoms treated by a mixture of independent and constrained refinement
Δρ_max_ = 0.20 e Å^−3^
Δρ_min_ = −0.28 e Å^−3^

### 

Data collection: *CrysAlis CCD* (Oxford Diffraction, 2009 ▶); cell refinement: *CrysAlis RED* (Oxford Diffraction, 2009 ▶); data reduction: *CrysAlis RED*; program(s) used to solve structure: *SHELXS97* (Sheldrick, 2008 ▶); program(s) used to refine structure: *SHELXL97* (Sheldrick, 2008 ▶); molecular graphics: *PLATON* (Spek, 2009 ▶); software used to prepare material for publication: *SHELXL97*.

## Supplementary Material

Crystal structure: contains datablocks I, global. DOI: 10.1107/S1600536810016235/bt5261sup1.cif

Structure factors: contains datablocks I. DOI: 10.1107/S1600536810016235/bt5261Isup2.hkl

Additional supplementary materials:  crystallographic information; 3D view; checkCIF report

## Figures and Tables

**Table 1:** Hydrogen-bond geometry (Å, °)

*D*—H⋯*A*	*D*—H	H⋯*A*	*D*⋯*A*	*D*—H⋯*A*
N1—H1*N*⋯O2^i^	0.82 (2)	2.16 (2)	2.974 (2)	176 (2)

Symmetry code: (i) 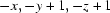.

**Fig. 1.**
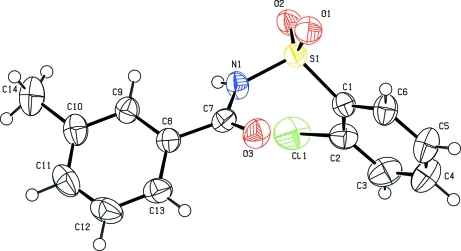
Molecular structure of the title compound, showing the atom-labelling scheme. Displacement ellipsoids are drawn at the 50% probability level.

**Fig. 2.**
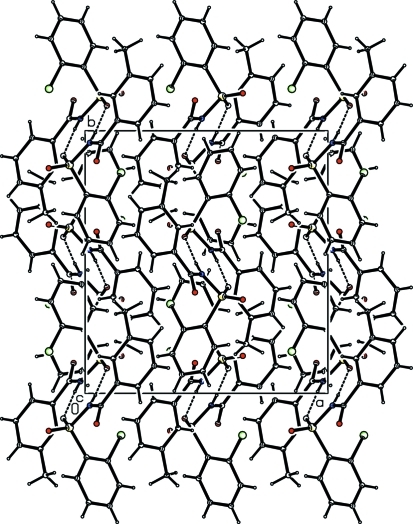
Molecular packing in the title compound. Hydrogen bonds are shown as dashed lines.

## supplementary crystallographic information

### Comment

As a part of studying the effect of ring and the side chain substitutions on the crystal structures of *N*-aryl sulfonamides (Gowda *et al.*, 2009, 2010; Suchetan *et al.*, 2010)), in the present work, the structure of 2-chloro-*N*-(3-methylbenzoyl)benzenesulfonamide, (I), has been determined. In the C—SO_2_—NH—C(O) segment, the N—H bond is *anti* to the C=O bond (Fig.1), similar to those observed in 2-chloro-*N*-(2-methylbenzoyl)benzenesulfonamide (II) (Suchetan *et al.*, 2010), *N*-(benzoyl)benzenesulfonamide (III) (Gowda *et al.*, 2009), and *N*-(benzoyl)2-chlorobenzenesulfonamide (IV) (Gowda *et al.*, 2010).

Further, the conformation of the C=O bond in the C—SO_2_—NH—C(O) segment of (I) is *anti* to the *meta*-methyl group in the benzoyl ring, contrary to the *syn* conformation observed between the *ortho*-methyl group and the C=O bond in (II).

The molecules are twisted at the *S* atom with the torsional angle of -66.5 (2)°, compared to those of -64.0 (2)° in (II), -66.9 (3)° in (III), and 66.7 (2)° in (IV).

The dihedral angle between the sulfonyl benzene ring and the —SO_2_—NH—C—O segment is 88.4 (1)°, compared to the values of 84.8 (1)° in (II), 86.5 (1) in (III) and 87.3 (1)° in (IV). Furthermore, the dihedral angle between the sulfonyl and the benzoyl benzene rings is 74.7 (1)°, compared to the values of 78.7 (1)° in (II), of 80.3 (1) in (III) and 73.3 (1)° in (IV).

The packing of molecules linked by N—H···O hydrogen bonds (Table 1) is shown in Fig. 2.

### Experimental

The title compound was prepared by refluxing a mixture of 3-methylbenzoic acid, 2-chlorobenzenesulfonamide and phosphorous oxy chloride for 3 h on a water bath. The resultant mixture was cooled and poured into ice cold water. The solid obtained was filtered, washed thoroughly with water and then dissolved in sodium bicarbonate solution. The compound was later reprecipitated by acidifying the filtered solution with dilute HCl. It was filtered, dried and recrystallized.

Prism like colourless single crystals of the title compound used in X-ray diffraction studies were obtained by slow evaporation of its toluene solution at room temperature.

### Refinement

The H atom of the NH group was located in a difference map and refined with a distance  restraint of N—H = 0.86 (2)Å. The other H atoms were positioned with idealized geometry and refined using a riding model with C—H = 0.93–0.96 Å. All H atoms were refined with isotropic displacement parameters  set to 1.2 times of the *U*_eq_ of the parent atom.

### Crystal data

**Table:**

C_14_H_12_ClNO_3_S	*F*(000) = 1280
*M**_r_* = 309.76	*D*_x_ = 1.407 Mg m^−^^3^
Orthorhombic, *P**b**c**a*	Mo *K*α radiation, λ = 0.71073 Å
Hall symbol: -P 2ac 2ab	Cell parameters from 5313 reflections
*a* = 12.4487 (8) Å	θ = 2.8–27.7°
*b* = 13.4619 (8) Å	µ = 0.41 mm^−^^1^
*c* = 17.455 (1) Å	*T* = 299 K
*V* = 2925.2 (3) Å^3^	Prism, colourless
*Z* = 8	0.30 × 0.30 × 0.20 mm

### Data collection

**Table:**

Oxford Diffraction Xcalibur diffractometer with a Sapphire CCD detector	2955 independent reflections
Radiation source: fine-focus sealed tube	2422 reflections with *I* > 2σ(*I*)
graphite	*R*_int_ = 0.014
Rotation method data acquisition using ω and phi scans	θ_max_ = 26.4°, θ_min_ = 2.9°
Absorption correction: multi-scan (*CrysAlis RED*; Oxford Diffraction, 2009)	*h* = −15→11
*T*_min_ = 0.887, *T*_max_ = 0.923	*k* = −16→9
7216 measured reflections	*l* = −12→21

### Refinement

**Table:**

Refinement on *F*^2^	Secondary atom site location: difference Fourier map
Least-squares matrix: full	Hydrogen site location: inferred from neighbouring sites
*R*[*F*^2^ > 2σ(*F*^2^)] = 0.035	H atoms treated by a mixture of independent and constrained refinement
*wR*(*F*^2^) = 0.095	*w* = 1/[σ^2^(*F*_o_^2^) + (0.0429*P*)^2^ + 1.518*P*] where *P* = (*F*_o_^2^ + 2*F*_c_^2^)/3
*S* = 1.04	(Δ/σ)_max_ = 0.001
2955 reflections	Δρ_max_ = 0.20 e Å^−^^3^
186 parameters	Δρ_min_ = −0.28 e Å^−^^3^
1 restraint	Extinction correction: *SHELXL97* (Sheldrick, 2008), Fc^*^=kFc[1+0.001xFc^2^λ^3^/sin(2θ)]^-1/4^
Primary atom site location: structure-invariant direct methods	Extinction coefficient: 0.0265 (11)

### Special details

**Table:**

Experimental. CrysAlis RED (Oxford Diffraction, 2009) Empirical absorption correction using spherical harmonics, implemented in SCALE3 ABSPACK scaling algorithm.
Geometry. All esds (except the esd in the dihedral angle between two l.s. planes) are estimated using the full covariance matrix. The cell esds are taken into account individually in the estimation of esds in distances, angles and torsion angles; correlations between esds in cell parameters are only used when they are defined by crystal symmetry. An approximate (isotropic) treatment of cell esds is used for estimating esds involving l.s. planes.
Refinement. Refinement of *F*^2^ against ALL reflections. The weighted *R*-factor wR and goodness of fit *S* are based on *F*^2^, conventional *R*-factors *R* are based on *F*, with *F* set to zero for negative *F*^2^. The threshold expression of *F*^2^ > σ(*F*^2^) is used only for calculating *R*-factors(gt) etc. and is not relevant to the choice of reflections for refinement. *R*-factors based on *F*^2^ are statistically about twice as large as those based on *F*, and *R*- factors based on ALL data will be even larger.

### Fractional atomic coordinates and isotropic or equivalent isotropic displacement parameters (Å^2^)

**Table:**

	*x*	*y*	*z*	*U*_iso_*/*U*_eq_	
C1	−0.00620 (14)	0.25666 (13)	0.42487 (10)	0.0366 (4)	
C2	−0.09360 (15)	0.24307 (16)	0.47300 (11)	0.0452 (5)	
C3	−0.1408 (2)	0.15010 (19)	0.47939 (15)	0.0634 (6)	
H3	−0.1992	0.1408	0.5118	0.076*	
C4	−0.1008 (2)	0.07177 (18)	0.43768 (16)	0.0688 (7)	
H4	−0.1323	0.0095	0.4423	0.083*	
C5	−0.0152 (2)	0.08419 (16)	0.38944 (15)	0.0627 (6)	
H5	0.0109	0.0307	0.3614	0.075*	
C6	0.03249 (18)	0.17676 (14)	0.38256 (12)	0.0482 (5)	
H6	0.0904	0.1855	0.3496	0.058*	
C7	−0.06413 (15)	0.45303 (13)	0.31212 (10)	0.0381 (4)	
C8	−0.13360 (15)	0.53961 (13)	0.29432 (10)	0.0373 (4)	
C9	−0.11657 (16)	0.63233 (13)	0.32775 (11)	0.0404 (4)	
H9	−0.0593	0.6410	0.3613	0.049*	
C10	−0.18376 (18)	0.71203 (15)	0.31173 (11)	0.0476 (5)	
C11	−0.26884 (18)	0.69666 (18)	0.26178 (13)	0.0581 (6)	
H11	−0.3156	0.7487	0.2510	0.070*	
C12	−0.28515 (18)	0.60527 (19)	0.22792 (13)	0.0598 (6)	
H12	−0.3426	0.5966	0.1946	0.072*	
C13	−0.21746 (16)	0.52658 (15)	0.24283 (11)	0.0484 (5)	
H13	−0.2278	0.4657	0.2188	0.058*	
C14	−0.1632 (2)	0.81144 (17)	0.34798 (15)	0.0693 (7)	
H14A	−0.1193	0.8509	0.3145	0.083*	
H14B	−0.1267	0.8023	0.3959	0.083*	
H14C	−0.2303	0.8447	0.3567	0.083*	
N1	−0.01969 (13)	0.45407 (11)	0.38535 (9)	0.0409 (4)	
H1N	−0.0421 (17)	0.4933 (14)	0.4174 (11)	0.049*	
O1	0.14949 (11)	0.35731 (10)	0.36476 (9)	0.0497 (4)	
O2	0.08974 (11)	0.40436 (10)	0.49378 (8)	0.0465 (4)	
O3	−0.04744 (13)	0.38456 (10)	0.26879 (8)	0.0528 (4)	
Cl1	−0.14827 (5)	0.34034 (5)	0.52560 (4)	0.0706 (2)	
S1	0.06458 (4)	0.37028 (3)	0.41809 (3)	0.03622 (15)	

### Atomic displacement parameters (Å^2^)

**Table:**

	*U*^11^	*U*^22^	*U*^33^	*U*^12^	*U*^13^	*U*^23^
C1	0.0387 (9)	0.0376 (9)	0.0335 (9)	0.0023 (8)	0.0006 (7)	0.0023 (7)
C2	0.0403 (10)	0.0532 (11)	0.0422 (10)	−0.0003 (9)	0.0050 (8)	−0.0039 (9)
C3	0.0539 (13)	0.0707 (15)	0.0656 (15)	−0.0153 (12)	0.0158 (12)	0.0052 (12)
C4	0.0806 (17)	0.0468 (12)	0.0789 (17)	−0.0165 (12)	0.0058 (14)	0.0043 (12)
C5	0.0829 (17)	0.0386 (11)	0.0665 (15)	0.0010 (11)	0.0102 (13)	−0.0030 (10)
C6	0.0581 (12)	0.0400 (10)	0.0464 (11)	0.0047 (9)	0.0100 (10)	0.0002 (9)
C7	0.0421 (10)	0.0362 (9)	0.0362 (9)	−0.0064 (8)	−0.0011 (8)	0.0008 (7)
C8	0.0403 (10)	0.0395 (9)	0.0321 (9)	−0.0028 (8)	−0.0009 (7)	0.0073 (7)
C9	0.0469 (10)	0.0395 (9)	0.0350 (9)	−0.0003 (8)	0.0001 (8)	0.0052 (7)
C10	0.0554 (12)	0.0446 (10)	0.0428 (10)	0.0075 (9)	0.0127 (9)	0.0098 (9)
C11	0.0504 (13)	0.0652 (13)	0.0587 (13)	0.0154 (11)	0.0049 (10)	0.0234 (11)
C12	0.0463 (12)	0.0789 (16)	0.0540 (13)	−0.0044 (11)	−0.0136 (10)	0.0220 (12)
C13	0.0500 (11)	0.0536 (11)	0.0417 (10)	−0.0112 (9)	−0.0079 (9)	0.0090 (9)
C14	0.0911 (18)	0.0452 (12)	0.0716 (16)	0.0147 (12)	0.0123 (14)	0.0024 (11)
N1	0.0514 (9)	0.0365 (8)	0.0349 (8)	0.0099 (7)	−0.0053 (7)	−0.0028 (6)
O1	0.0420 (7)	0.0497 (8)	0.0576 (9)	−0.0001 (6)	0.0114 (7)	0.0031 (7)
O2	0.0493 (8)	0.0466 (7)	0.0438 (8)	0.0065 (6)	−0.0130 (6)	−0.0061 (6)
O3	0.0656 (10)	0.0463 (8)	0.0464 (8)	0.0020 (7)	−0.0051 (7)	−0.0112 (6)
Cl1	0.0563 (4)	0.0847 (4)	0.0708 (4)	0.0028 (3)	0.0242 (3)	−0.0256 (3)
S1	0.0360 (2)	0.0358 (2)	0.0368 (3)	0.00304 (18)	−0.00100 (18)	−0.00045 (18)

### Geometric parameters (Å, °)

**Table:**

C1—C2	1.387 (3)	C9—C10	1.389 (3)
C1—C6	1.391 (3)	C9—H9	0.9300
C1—S1	1.7691 (18)	C10—C11	1.387 (3)
C2—C3	1.387 (3)	C10—C14	1.502 (3)
C2—Cl1	1.738 (2)	C11—C12	1.380 (3)
C3—C4	1.375 (4)	C11—H11	0.9300
C3—H3	0.9300	C12—C13	1.378 (3)
C4—C5	1.368 (3)	C12—H12	0.9300
C4—H4	0.9300	C13—H13	0.9300
C5—C6	1.385 (3)	C14—H14A	0.9600
C5—H5	0.9300	C14—H14B	0.9600
C6—H6	0.9300	C14—H14C	0.9600
C7—O3	1.210 (2)	N1—S1	1.6430 (16)
C7—N1	1.393 (2)	N1—H1N	0.819 (15)
C7—C8	1.484 (3)	O1—S1	1.4192 (14)
C8—C13	1.389 (3)	O2—S1	1.4333 (14)
C8—C9	1.394 (3)		
			
C2—C1—C6	119.43 (18)	C11—C10—C9	118.1 (2)
C2—C1—S1	123.05 (14)	C11—C10—C14	121.9 (2)
C6—C1—S1	117.43 (14)	C9—C10—C14	120.0 (2)
C1—C2—C3	119.99 (19)	C12—C11—C10	121.0 (2)
C1—C2—Cl1	121.86 (16)	C12—C11—H11	119.5
C3—C2—Cl1	118.14 (16)	C10—C11—H11	119.5
C4—C3—C2	119.8 (2)	C13—C12—C11	121.0 (2)
C4—C3—H3	120.1	C13—C12—H12	119.5
C2—C3—H3	120.1	C11—C12—H12	119.5
C5—C4—C3	120.9 (2)	C12—C13—C8	119.0 (2)
C5—C4—H4	119.5	C12—C13—H13	120.5
C3—C4—H4	119.5	C8—C13—H13	120.5
C4—C5—C6	119.8 (2)	C10—C14—H14A	109.5
C4—C5—H5	120.1	C10—C14—H14B	109.5
C6—C5—H5	120.1	H14A—C14—H14B	109.5
C5—C6—C1	120.1 (2)	C10—C14—H14C	109.5
C5—C6—H6	120.0	H14A—C14—H14C	109.5
C1—C6—H6	120.0	H14B—C14—H14C	109.5
O3—C7—N1	120.86 (17)	C7—N1—S1	124.46 (13)
O3—C7—C8	124.56 (17)	C7—N1—H1N	119.9 (15)
N1—C7—C8	114.55 (16)	S1—N1—H1N	115.0 (15)
C13—C8—C9	119.89 (18)	O1—S1—O2	118.76 (9)
C13—C8—C7	118.31 (17)	O1—S1—N1	109.39 (9)
C9—C8—C7	121.80 (16)	O2—S1—N1	103.91 (8)
C10—C9—C8	121.05 (18)	O1—S1—C1	107.97 (8)
C10—C9—H9	119.5	O2—S1—C1	108.90 (8)
C8—C9—H9	119.5	N1—S1—C1	107.39 (8)
			
C6—C1—C2—C3	0.8 (3)	C8—C9—C10—C14	−179.49 (19)
S1—C1—C2—C3	−175.56 (17)	C9—C10—C11—C12	−1.1 (3)
C6—C1—C2—Cl1	−178.29 (16)	C14—C10—C11—C12	178.7 (2)
S1—C1—C2—Cl1	5.3 (2)	C10—C11—C12—C13	0.1 (3)
C1—C2—C3—C4	−0.2 (4)	C11—C12—C13—C8	1.7 (3)
Cl1—C2—C3—C4	178.9 (2)	C9—C8—C13—C12	−2.5 (3)
C2—C3—C4—C5	−0.3 (4)	C7—C8—C13—C12	177.57 (18)
C3—C4—C5—C6	0.3 (4)	O3—C7—N1—S1	4.7 (3)
C4—C5—C6—C1	0.4 (4)	C8—C7—N1—S1	−176.87 (13)
C2—C1—C6—C5	−0.9 (3)	C7—N1—S1—O1	50.47 (18)
S1—C1—C6—C5	175.69 (18)	C7—N1—S1—O2	178.25 (15)
O3—C7—C8—C13	27.9 (3)	C7—N1—S1—C1	−66.46 (17)
N1—C7—C8—C13	−150.47 (17)	C2—C1—S1—O1	179.10 (16)
O3—C7—C8—C9	−152.07 (19)	C6—C1—S1—O1	2.64 (18)
N1—C7—C8—C9	29.6 (2)	C2—C1—S1—O2	48.88 (18)
C13—C8—C9—C10	1.4 (3)	C6—C1—S1—O2	−127.58 (15)
C7—C8—C9—C10	−178.59 (17)	C2—C1—S1—N1	−63.04 (18)
C8—C9—C10—C11	0.4 (3)	C6—C1—S1—N1	120.49 (16)

### Hydrogen-bond geometry (Å, °)

**Table:**

*D*—H···*A*	*D*—H	H···*A*	*D*···*A*	*D*—H···*A*
N1—H1N···O2^i^	0.82 (2)	2.16 (2)	2.974 (2)	176 (2)

Symmetry codes: (i) −*x*, −*y*+1, −*z*+1.

## References

[bb1] Gowda, B. T., Foro, S., Suchetan, P. A. & Fuess, H. (2009). *Acta Cryst.* E**65**, o2516. 10.1107/S1600536809037222 PMC2970249 21577963

[bb2] Gowda, B. T., Foro, S., Suchetan, P. A. & Fuess, H. (2010). *Acta Cryst.* E**66**, o794. 10.1107/S1600536810008731 PMC2984022 21580633

[bb3] Oxford Diffraction (2009). *CrysAlis CCD* and *CrysAlis RED* Oxford Diffraction Ltd, Yarnton, Oxfordshire, England.

[bb4] Sheldrick, G. M. (2008). *Acta Cryst.* A**64**, 112–122. 10.1107/S0108767307043930 18156677

[bb5] Spek, A. L. (2009). *Acta Cryst.* D**65**, 148–155. 10.1107/S090744490804362X PMC2631630 19171970

[bb6] Suchetan, P. A., Gowda, B. T., Foro, S. & Fuess, H. (2010). *Acta Cryst.* E**66**, o1281. 10.1107/S1600536810015990 PMC2979549 21579380

